# Smart Kiosk for Nutritional Management of People With Diabetes in Underserved Communities: Development and Technical Evaluation

**DOI:** 10.2196/76936

**Published:** 2026-02-19

**Authors:** Guadalupe Esmeralda Rivera-García, Juan Carlos Ramírez-Vázquez, Jaime Cruz-Casados, Miriam Janet Cervantes-López, Arturo LLanes-Castillo, Marco Antonio Diaz-Martinez

**Affiliations:** 1 Tecnológico Nacional de México, Instituto Tecnológico Superior de Pánuco, Av. Artículo Tercero Constitucional, Colonia Solidaridad, Pánuco, Veracruz, 93990, Mexico, 52 846-106-4254; 2 Facultad de Medicina de Tampico “Dr. Alberto Romo Caballero”, Universidad Autónoma de Tamaulipas, Tampico, Tamaulipas, Mexico

**Keywords:** smart health kiosk, diabetes management, nutritional recommendation system, artificial intelligence, underserved communities

## Abstract

**Background:**

Diabetes is a chronic disease with a high global prevalence, increasing from 200 million people in 1990 to 830 million in 2022, with a higher burden in low- and middle-income regions and high mortality in Mexico and Veracruz. These inequalities limit access to treatment and nutritional education, requiring technological solutions such as interactive kiosks based on artificial intelligence (AI) that contribute to the nutritional management of people with diabetes in marginalized communities.

**Objective:**

This study aimed to design and evaluate an interactive kiosk based on AI that generates culturally relevant and personalized meal plans for people with diabetes in marginalized communities.

**Methods:**

A low-cost prototype was developed, with a database of local foods and a multilayer perceptron trained with synthetic data based on national clinical guidelines. Performance was tested through an experimental evaluation that measured (1) the accuracy of nutritional recommendations compared with ideal meal plans (accuracy, precision, sensitivity, and *F*_1_-score); (2) performance, measured by recording response time with 1 to 50 simultaneous requests; and (3) usability, assessed using heuristic evaluation and the System Usability Scale (SUS).

**Results:**

The smart kiosk was experimentally evaluated in three dimensions: nutritional recommendations, system efficiency, and usability. The model achieved AI metrics of 87.3% overall accuracy, 90.5% precision, 92.1% sensitivity, and 91.3% *F*_1_-score. The average response time was 2.36 (SD 0.24) seconds in all load tests. A maximum time of 4 seconds was obtained in the simulation of 50 concurrent users. In the usability evaluation, an average score of 89 (SD 2.89) out of 100 was obtained on the SUS, which is considered excellent, along with a success rate of 98.3%.

**Conclusions:**

The AI-based kiosk demonstrated technical feasibility, adequate performance, and satisfactory usability. Its ability to operate without the need for internet and its low cost make it an equitable option for diabetes self-management and a replicable model in public health.

## Introduction

Diabetes mellitus is a global public health problem. In 1990, it affected approximately 200 million people; by 2022, this number had surpassed 830 million. This increase has occurred primarily in low- and middle-income countries, where more than half of those with the disease are not receiving treatment [1]. The outlook is grim: by 2050, diabetes mortality is projected to increase by 59.7%, reaching 1.31 billion cases [2]. In Mexico, cases are also on the rise; prevalence increased from 16.8% in 2018 [3] to 18.3% in 2022 [4].

Diabetes is not a biological disease; it is a social one. There is a well-established gradient in which the poorest people become ill more frequently and die earlier [5]. Veracruz, for example, was the second poorest state in the country in 2022, with 4.2 million people living in poverty and almost half of its population lacking access to health care [6]. In the municipality of Pánuco, where the interactive kiosk evaluated in this study is planned, 48.9% of the population lived in poverty in 2020 [7], and there was only one physician for every 1000 inhabitants [8].

Diabetes management requires comprehensive self-care strategies and nutritional education, as proper nutrition and the adoption of healthy lifestyles are fundamental to preventing complications [9].

In this context, artificial intelligence (AI) has become relevant in health care, as it can process large amounts of clinical data and aid in decision-making, and the market for AI-powered health technologies is growing rapidly [10,11]. In response to this scenario, this project aims to develop a low-cost, culturally adapted, offline-operating smart kiosk with a local database and a user-friendly interface for people with limited digital literacy. These technologies, developed primarily in community and low-resource settings, promote digital inclusion and equitable access to health care in nonhospital settings [12,13]. Evidence exists for the use of health kiosks for chronic disease monitoring [14,15] and their effectiveness in nutritional education and glycemic control [16-18].

In this context, this study aimed to develop and test an AI-powered interactive kiosk that can provide personalized nutritional recommendations to people with diabetes in vulnerable communities. The device was designed to be autonomous, requiring no on-site health care personnel, and to provide understandable information on nutrition and individualized meal plans. Its effectiveness was evaluated quantitatively and experimentally in terms of accuracy, efficiency, and ease of use.

## Methods

### Overall Research Design

This study adopted a quantitative approach with an experimental design to verify the technical performance of the AI-based interactive kiosk before its public launch. The accuracy of the nutritional recommendations produced by the system was evaluated, as well as its performance and ease of use. To this end, an experimental evaluation was carried out with predefined test data that simulated real-life usage scenarios. The working hypothesis was that the intelligent system could (1) generate personalized nutritional recommendations with an accuracy level greater than or equal to 85%, based on ideal expert recommendations; (2) maintain adequate performance (response times of less than 3 seconds per query); and (3) meet basic usability criteria (high task success rate and low user error rate). On the basis of this hypothesis, quantitative metrics and acceptance thresholds were established in accordance with software engineering standards. The entire process was structured into three main phases: (1) development of the AI-based interactive kiosk, (2) training and validation of the AI model, and (3) experimental evaluation of the system.

### Phase 1: Development of the AI-Based Interactive Kiosk

#### Overview

In this phase, the kiosk infrastructure was built, both in terms of software and the user interface (UI), and the AI engine responsible for generating recommendations was integrated. An agile incremental methodology was adopted, which allowed for iterative progress and continuous validation of each component. In addition, contributions were incorporated from 3 nutrition specialists from the Autonomous University of Tamaulipas, Tampico, Tamaulipas, and 3 physicians in computer systems specializing in human-computer interaction from the Higher Technological Institute of Pánuco, Veracruz. The design is based on three principles: low cost, offline operation, and cultural relevance in a regional context. The system can be synchronized with a remote server to store or update data when connectivity is available, thereby ensuring data integrity.

The following sections describe (1) the architecture and components of the kiosk, (2) the UI, (3) the AI module, (4) the nutritional database, (5) the synchronization or server component (optional), (6) data initiation and recording, (7) processing and generation of recommendations, (8) presentation of results to the user, (9) the interactive education section, and (10) system shutdown and additional options.

#### Kiosk Architecture and Components

**Figure 1.**
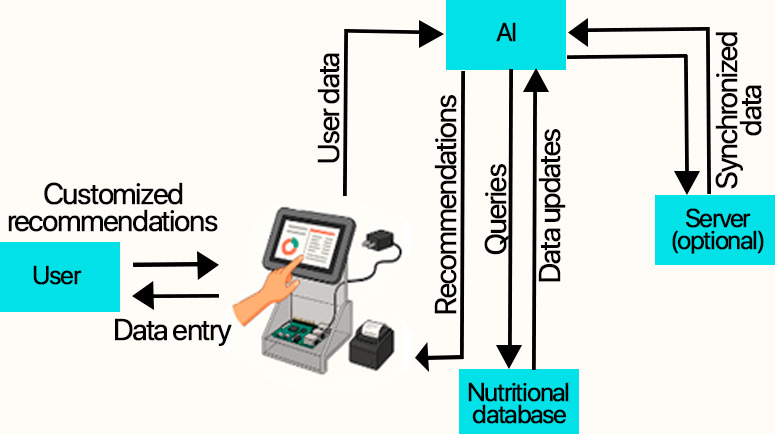
Architecture of the artificial intelligence (AI)–based interactive kiosk: diagram showing the process for the user to receive nutritional recommendations.

The interactive kiosk was designed as a comprehensive solution consisting of various software modules integrated into a low-cost hardware platform, designed to operate autonomously in marginalized communities. The elements that make up the kiosk’s hardware are a Raspberry Pi 4B (4 GB RAM), a 10.1” HDMI touchscreen, a USB audio module, a 5V/3A power supply, an acrylic structure, and a mini thermal printer. Its architecture allows users to interact via a touchscreen, enter personal data, and receive nutritional recommendations generated by a neural network that consults a specialized database. Optionally, the AI model can be synchronized with a remote server to store data or receive updates. In terms of security, Transport Layer Security and HTTP Secure data encryption and token authentication are used (Figure 1).

#### User Interface

The interface was developed as a local web application, specifically designed for use by users with low literacy levels. It uses touch controls, large buttons, intuitive pictograms, and a minimalist design. It runs in full-screen mode using an integrated browser. In addition to Spanish text, it includes an audio system that reads instructions and recommendations, with the aim of facilitating use by people with reading difficulties. All these features help the user minimize typing errors when entering data. The process begins with the user entering data, calculating energy requirements using conventional equations (modified Harris-Benedict), and running the AI model, which generates the optimal menu based on dietary and metabolic restrictions. Finally, the meal plan is displayed on the screen and can be downloaded via a QR code or as a PDF file, or printed.

#### AI Module (Recommendation Engine)

The AI engine represents the core of the system and is programmed in Python using TensorFlow version 2.12 (Google). On the basis of the information provided by the user, the system calculates the BMI and estimates daily calorie requirements. It then generates a personalized nutrition plan that includes a distribution of calories by macronutrients, portions by food group, and examples of specific dishes.

#### Nutritional Database

This local database, developed with SQLite, stores detailed nutritional information on foods and recipes typical of the region, including dishes of cultural significance. It also incorporates dietary rules that allow the AI engine to adjust recommendations according to the patient’s profile. When generating suggestions, the system selects food combinations that meet caloric and macronutrient requirements, prioritizing those suitable for people with diabetes. Its offline functionality is achieved through real-time SQL queries. The main sources of information were NOM-015-SSA2-2023, NOM-043-SSA2-2012, NOM-051-SCFI/SSA1-2010, NOM-008-SSA3-2017, the Mexican Food Composition Tables (INCMNSZ, 2020), local community recipe books, and national clinical nutrition guidelines for diabetes (Centro Nacional de Excelencia Tecnológica en Salud), ensuring the technical validity and cultural adaptation of the system.

#### Synchronization Component and Server (Optional)

Although the kiosk is designed to operate offline, a synchronization module was included that allows, when connected, to send usage statistics to a central server and receive system updates. This functionality was developed in Python and allows the system to be kept up to date with new prescriptions or changes in medical guidelines. Communication with the server is carried out using Transport Layer Security (TLS) and HTTPS, with token-based authentication to ensure information security during the synchronization process.

#### Processing and Generation of the Recommendation

**Figure 2.**
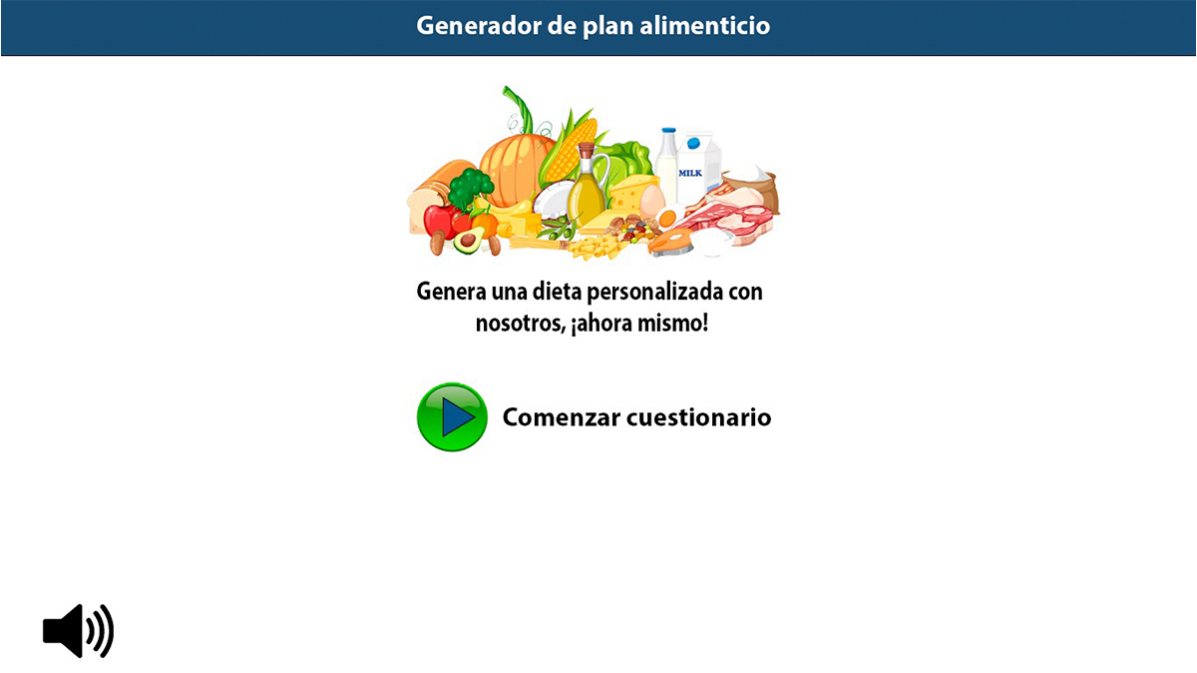
Interactive kiosk home screen displaying the icon to start a questionnaire where the user’s personal and clinical data are entered, which is automatically validated before processing. It also has an audio system to repeat the instructions.

When logging in, users must fill out a questionnaire with information such as age, weight, type of diabetes, level of physical activity, and dietary preferences. The system verifies the information entered using type and range checks, predefined fields, internal consistency rules, and semantic validation to avoid errors, ensuring that the data provided are consistent and suitable for processing (Figure 2).

**Figure 3.**
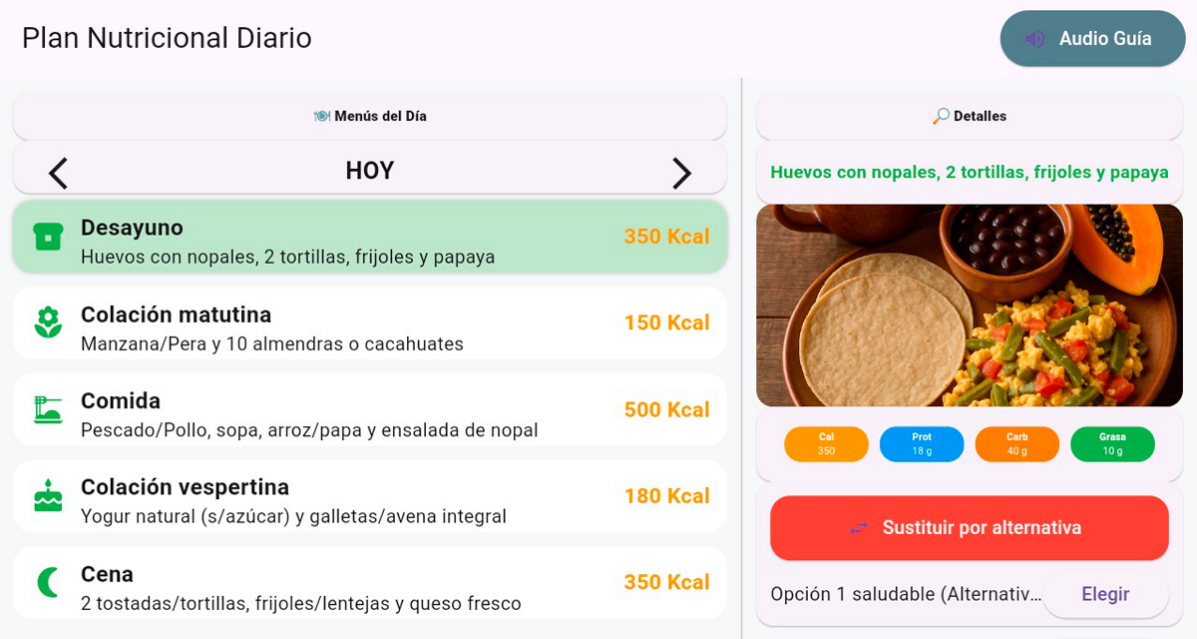
Presentation of the nutritional plan with content that combines simple texts, images, and explanatory audio, allowing users to better understand the recommendations proposed.

Once the data have been entered and verified, the system begins processing the recommendation. The AI engine calculates key indicators such as the BMI and establishes individual caloric requirements. With this information, it generates a personalized meal plan tailored to the user’s profile. The nutritional plan is presented on the screen in a visual and organized manner: it includes a calorie summary and menus for each meal time. The user can explore each section, review the nutritional information for each dish, and even substitute foods for equivalent alternatives. At the end, the user has the option to print a summary of their plan or store their information using an alias or a QR code for future sessions. The average duration of each session is 5 minutes. The system is designed to automatically reset for the next user. Figure 3 shows an example of a nutritional plan generated by the kiosk.

### Phase 2: Training and Validation of the AI Model

The AI engine was developed in the Python programming language using the TensorFlow version 2.12 library.

A multilayer perceptron was designed for multiclass classification, trained with 10,000 synthetic data points. Data were based on Mexican clinical nutrition and diabetes standards and guidelines and the nutritional ranges recommended by Encuesta Nacional de Salud y Nutrición Continua (ENSANUT) 2022. The dataset was enriched with recipes and local dishes from northern Veracruz.

Energy ranges between 1200 and 2800 kcal/d and macronutrient distributions within acceptable ranges (carbohydrates, 45%‐65%; proteins, 10%‐35%; and fats, 20%‐35%) were defined.

The data were obtained by stratified random sampling, with controlled variation in anthropometric profiles and dietary patterns. Each entry coded 15 informative variables that capture demographic, clinical, and nutritional aspects, such as diagnosis, level of physical activity, caloric needs, macronutrient distribution, predominant food group, and dietary adherence.

To maintain population realism, the proportions of each stratum were scaled to the distribution in the Mexican population with type 2 diabetes according to ENSANUT 2022. Values outside defined physiological ranges (eg, BMI <15 kg/m² or >40 kg/m²) were automatically removed, and nutritional consistency filters were applied. The final dataset was exported in CSV format to train and validate the multilayer neural network model in an 80% and 20% partition scheme (training and testing).

The model consists of 3 hidden layers of 64 neurons with rectified linear unit activation. Batch normalization was also implemented, along with a dropout technique with a rate of 0.3 to prevent overfitting.

The output layer consists of three neurons with softmax activation, corresponding to the different classes: “suitable,” “minor adjustment,” and “consultation required.” The model was trained using the Adam optimizer (learning rate=0.001) and the categorical cross-entropy loss function, incorporating an early stopping criterion when the validation loss stopped improving or after reaching 100 epochs. The data were divided as follows: training (80%) and validation (20%), and to improve the generalization of the model, L2 regularization was applied. The model used variables such as age, sex, height, weight, physical activity, type of diabetes, glycemic control, and food preferences to calculate BMI and calorie requirements. A deterministic submodule based on nutritional heuristic rules generates a personalized meal plan, prioritizing low-glycemic and high-fiber foods. The result is provided in an interactive graphical UI that displays a summary of calories, macronutrients, and menus by meal times, in which equivalent foods can be substituted to allow flexibility and improve adherence to the diet.

### Phase 3: Experimental Evaluation of the System

#### Overview

Once the prototype was built, quantitative tests were performed. These tests were classified into three categories: (1) performance of the AI model in generating nutritional recommendations, (2) system performance and efficiency (response times and resource usage), and (3) interface usability.

#### Performance Testing of the AI Model in Nutritional Recommendations

A test dataset was defined that covered the essential functionalities of user data recording, nutritional recommendation generation, and educational information display. To evaluate performance, the recommendations issued by the system for each profile were compared with their reference equivalents, previously validated by nutritionists at the Autonomous University of Tamaulipas, Tampico, Tamaulipas. On the basis of this comparison, several indicators were calculated: overall agreement rate (accuracy), precision, sensitivity, defined as the ability to choose the right foods for each patient, and the *F*_1_-score. In addition, each suggestion was verified to comply with basic nutritional guidelines, such as not exceeding a specific carbohydrate threshold per meal for people with diabetes and including essential food groups. The test data were user registration, nutritional recommendations, and educational content.

#### System Performance and Efficiency Testing

The system response time was measured with variable loads. The time from when the user enters data to when the recommendation appears was recorded.

To ensure validity, each case was run several times, and response times were recorded to calculate mean values with their corresponding SD.

The scalability of the system was evaluated with loads of 5, 10, 20, and 50 users. For each test, the average response time, standard deviation (SD), and average CPU and RAM usage were recorded. The software was run offline to simulate the behavior of the system in a low-cost, resource-limited environment, such as the community spaces where it will operate. Performance tests were conducted on the kiosk's own hardware, simulating multiple concurrent users using threads.

#### Interface Usability Testing (Simulated Heuristic Evaluation)

As no real patients were involved, this evaluation focused on identifying interface design issues and determining whether the application was understandable and easy to use for the target population (adults with basic education). All data collected during testing were stored for subsequent statistical analysis. For each metric, mean values were calculated, and, where applicable, 95% CIs were estimated. As the study did not have a human control group, the analysis was enriched by a technical comparison between the AI-based recommendation engine and an alternative module based on fixed rules (without advanced personalization), using the same test profiles to quantify the improvement provided by AI. In addition, by using synthetic data and operating locally, the system guarantees user privacy, which eliminated the need for ethical approval. This methodology allowed for a comprehensive evaluation of the system in a controlled environment.

According to Nielsen and Molich [19], between 3 and 5 expert evaluators can detect most usability problems. Similarly, Sauro and Lewis [20] pointed out that 30 to 100 task executions are sufficient to obtain reliable metrics of success, error, and interaction time. Therefore, the 60 tasks evaluated by 3 experts were sufficient for this stage, as they fell within the suggested range.

### Ethical Considerations

In accordance with the Regulations of the General Health Law on Research and NOM-012-SSA3-2012, this study is not classified as research involving human participants: the tests were exclusively technical, and no personal data were collected, nor were any interventions performed on participants. This study did not involve experiments on humans or animals, nor did it involve the collection of sensitive personal data. It focused exclusively on the development and technical evaluation of the smart nutrition management kiosk. A controlled experiment was conducted with synthetic data (fictitious patients), and no private or identifiable information was accessed or stored; therefore, approval from an institutional review board was not required.

### Reporting Guidelines

This manuscript was prepared following the STARE-HI (Statement on Reporting of Evaluation Studies in Health Informatics) guidelines to ensure a clear and transparent description of the development and technical evaluation of the AI-based nutritional kiosk. A STARE-HI checklist is included as Checklist 1, indicating the section of the manuscript where each of the required criteria is addressed.

## Results

### Experimental Evaluation of the System

The results of the experimental evaluation of the system (phase 3) are presented. The tests and experiments carried out have enabled objective data to be collected on the operation of the smart kiosk. The findings are classified into three categories: (1) performance of the AI model in generating nutritional recommendations, (2) system performance and efficiency (response times and resource usage), and (3) usability of the interface.

### Performance of the AI Model in Nutritional Recommendations

Table 1 shows that the model achieved an overall accuracy of 87.3%, along with a precision of 90.5%, a sensitivity of 92.1%, and an F1-score of 91.3%. The results indicate that the model can distinguish between adequate meal plans and those that require review by a nutritionist, with a good balance between sensitivity and precision. The “Minor_Adjustment” class achieved 85.1% accuracy, 82.2% sensitivity, and an *F*_1_-score of 83.6%, which is slightly lower but still very consistent. On the other hand, the “Requires_Consultation” class obtained values above 80.0% in the 3 main metrics, which is an acceptable result considering the smaller number of samples in this class. On average, the macro average gave 85.2% accuracy, 84.8% sensitivity, and 85.0% *F*_1_-score, and the weighted average gave 87.3% in the 3 metrics, indicating good overall calibration of the model. These results indicate that the system strikes a good balance between coverage and precision, generalizing well across classes without significant bias. The performance and learning curves indicate that the synthetic set of approximately 10,000 records saturated the multilayer perceptron, with no improvements observed when increasing the training size, suggesting that it was sufficient for training.

**Table 1.** Performance metrics of the artificial intelligence–based nutritional recommendation model during the technical evaluation conducted in Veracruz, Mexico (2025).^a^

Class	TP^b^	FP^c^	FN^d^	Support	Precision (%)	Recall (%)	*F*_1_-score (%)
Adequate	152	16	13	165	90.5	92.1	91.3
Minor adjustment	74	13	16	90	85.1	82.2	83.6
Requires consultation	36	9	9	45	80.0	80.0	80.0
Macro average	—^e^	—	—	—	85.2	84.8	85.0
Weighted average	—	—	—	—	87.3	87.3	87.3
Micro average (overall accuracy)	—	—	—	—	87.3	87.3	87.3

a Metrics include class-level precision, recall, *F*_1_-score, and overall accuracy across 300 simulated patient profiles.

b TP: true positive.

c FP: false positive.

d FN: false negative.

e Not applicable.

### System Performance and Efficiency

**Figure 4.**
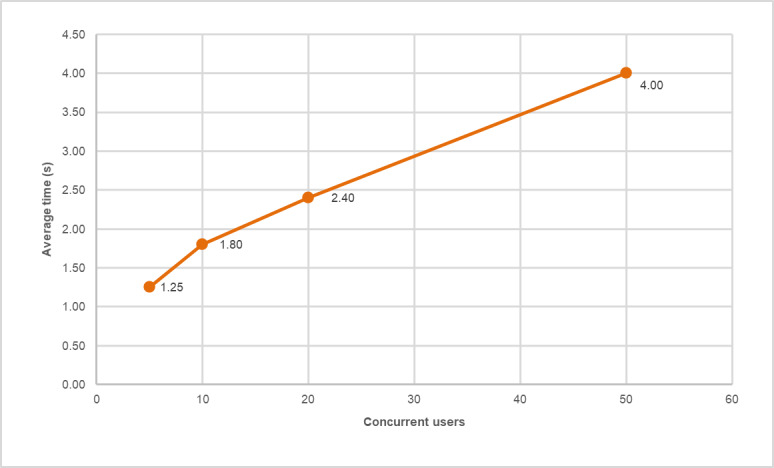
Scalability analysis of the interactive kiosk software during stress-testing simulations (2025). Average response time increased from 1.25 s (5 users) to 4.0 s (50 users), demonstrating a linear growth pattern under increasing computational demand.

Table 2 and Figure 4 summarize the main performance indicators of the smart kiosk software. The average response time was 2.36 (SD 0.24) seconds, with a maximum of 4 seconds under peak load (50 concurrent users). The SD of 0.24 seconds shows that the system consistently responds in a similar manner; there is little variation between requests.

**Table 2.** System performance and resource utilization of the artificial intelligence–based nutritional kiosk under simulated concurrent user loads (5‐50 users) during experimental testing in Veracruz, Mexico (2025).^a^

Concurrent users	Average time (s)	SD (s)	Average CPU (%)	Average RAM (%)
5	1.25	0.12	35.0	42.0
10	1.80	0.18	47.0	46.0
20	2.40	0.25	59.0	51.0
50	4.00	0.40	77.0	63.0
Total average	2.36	0.24	54.5	50.5

a Measures include average response time, CPU usage, and RAM consumption.

**Figure 5.**
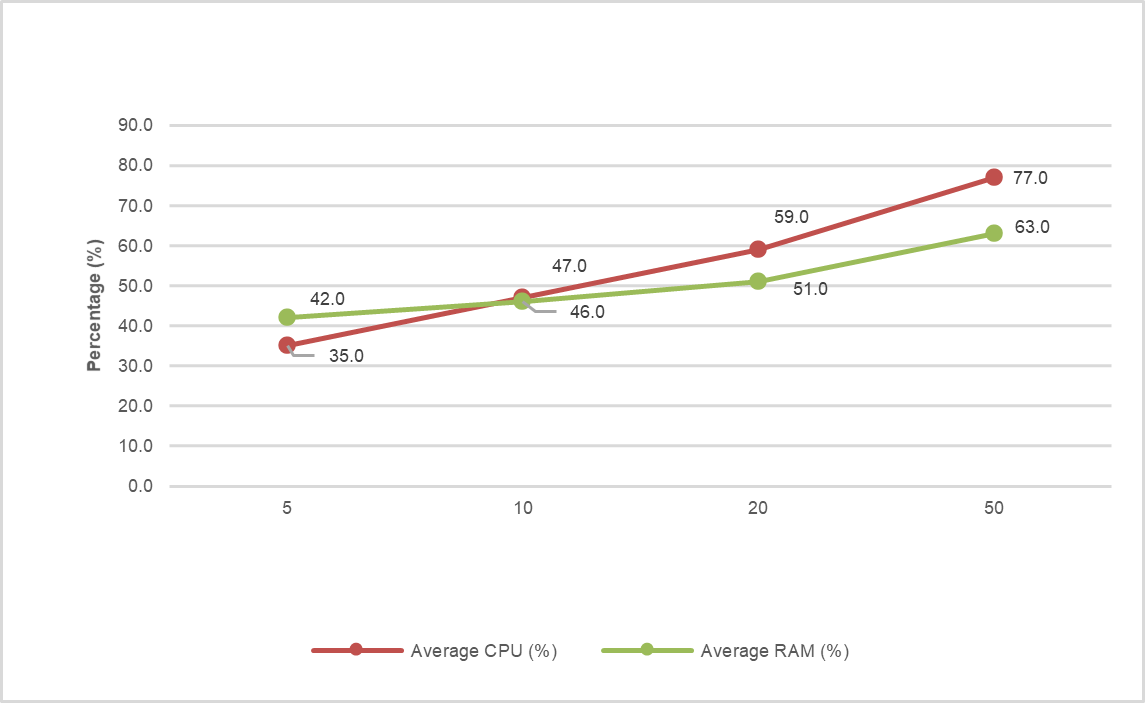
CPU and RAM utilization of the kiosk system during simulated load increments (5‐50 concurrent users) in the experimental evaluation (2025). Resource usage increased linearly without reaching saturation.

Resource usage remained at acceptable levels, with an average CPU usage of 54.5% and RAM usage of 50.5%, allowing the system to operate on embedded hardware or mid-range computers without compromising stability. The results demonstrate that the software core is stable, efficient, and scalable, making it suitable for environments with limited connectivity (Figure 5).

### Interface Usability

The combination of expert inspection and simulated testing provided valuable information on the kiosk’s ease of use. Overall, the 3 evaluators (PhDs in Computer Systems from the Higher Technological Institute of Pánuco, Veracruz) agreed that the interface is intuitive and accessible.

The evaluators highlighted the clear iconography, the legible font size for older adults, and the audio narration to guide the user. Among the improvements, they proposed adding a “back” button on all screens to correct input errors and highlighting foods “to avoid” in the summary, thus reinforcing critical information. No bugs or navigation problems were reported. The interface meets essential accessibility criteria: high color contrast, touch areas of at least 48 pixels to facilitate tapping, and audio playback for all important text, especially benefiting users with low vision or limited literacy.

Satisfaction was measured using the System Usability Scale (SUS) adapted to Spanish, with a Likert scale ranging from 1 (strongly disagree) to 5 (strongly agree). On average, the evaluators gave very high scores: ease of use, 5 out of 5; feature integration, 4 out of 5; confidence in independent use, 5 out of 5; and a low perceived need for prior learning, 2 out of 5. When these ratings were converted to the standardized SUS metric (0‐100), the interface achieved approximately 90 out of 100, placing it in the “excellent” usability range.

Table 3 presents that the average score for each evaluator (87.5%, 92.5%, and 87.5%), as well as the overall average score for the SUS, which was 89.17 (SD 2.89) out of 100, is classified as “excellent” (A). This score indicates that the system is usable, with an intuitive interface and easy navigation. The success rate was 98.3%, with a 95% CI, indicating that most actions were completed. The error rate, which was below 0.05, indicates the robustness of the interface and the clarity of the instructions. These indicators reveal that the smart kiosk software is highly usable.

**Table 3.** Usability evaluation results of the artificial intelligence–based nutritional kiosk obtained through heuristic expert testing (3 evaluators and 60 simulated tasks) performed in Veracruz, Mexico (2025).^a^

Indicator	Value
Participant 1 (%)	87.5
Participant 2 (%)	92.5
Participant 3 (%)	87.5
Average SUS^b^ score (0‐100; %)	89.17
Standard deviation (SUS score)	2.89
SUS classification	Excellent (grade A)
Task success rate, Wilson score (95% CI)	98.3% (Wilson) (91.1 %‐ 99.7 %)
Estimated error rate	<0.05

a Metrics include SUS score, task success rate, and estimated error rate.

b SUS: System Usability Scale.

## Discussion

### Comparison With Previous Work

Multiple studies have verified that educational interventions supported by digital technologies significantly improve glycemic control and self-care in people with diabetes [21-25]. Furthermore, AI has been able to predict events, manage chronic diseases, and provide personalized nutritional recommendations, with demonstrated improvements in treatment adherence and health outcomes when incorporated into structured interventions [26-39].

For type 2 diabetes, for example, various AI-powered apps have been developed for patient education, diagnostic support, clinical decision-making, and nutritional guidance, resulting in improved quality of life and more efficient care processes [40-61]. However, most of these solutions have not been implemented in low-resource clinical settings.

Given this reality, interactive health kiosks represent an innovative alternative for bringing health services to communities. Evidence supports their application in diagnostic support, telehealth, and health education, with growing international acceptance [62-70]. Furthermore, these devices have been useful for monitoring clinical variables and educating patients with diabetes, especially those with limited education or access to health care services [71-73].

Unlike previous research, this study proposes an AI-powered autonomous kiosk for underserved communities that does not require medical personnel or personal devices to reduce technological, economic, and educational barriers, thereby expanding nutrition and self-care education in vulnerable environments.

### Principal Findings

The results demonstrate that the AI-based interactive kiosk is not only technically feasible but also has the potential to improve nutritional management significantly. Controlled tests validated the accuracy of the recommendation engine, operational efficiency, and interface usability, laying the groundwork for its pilot implementation in real-world settings. The 87.3% agreement rate with plans designed by nutritionists shows that the neural network successfully learned the complex rules that experts apply when personalizing diets. This result is comparable to that achieved by initiatives such as PROTEIN in Europe, which also achieved expert-level automated recommendations [17]. Furthermore, the 92.1% sensitivity underscores that the kiosk incorporates almost all critical dietary components, thus minimizing the risk of nutritional deficiencies.

In addition, by incorporating local ingredients such as tortillas, nopales (prickly pear cactus), and beans, it aligns with the principles of precision nutrition and improves adherence, something rarely observed in studies focused on European or North American diets.

The average response time of 2.36 (SD 0.24) seconds ensures a smooth interactive experience, which is a critical factor in preventing early abandonment. This finding aligns with observations in health kiosks targeting African American communities, where interface speed was key to user acceptance among those with low literacy and limited patience [12]. Furthermore, the ability to operate for extended periods using low-cost hardware opens the door to its use in rural clinics, community centers, and mobile units.

Experts valued the clarity of the iconography, text size, and audio support elements recommended in the literature for rural and low-literacy settings [16]. A user-friendly interface not only facilitates initial adoption but also promotes repeated use, maximizing the health impact. While the kiosk can operate autonomously, its true potential emerges when integrated with the work of physicians and nutritionists. A hybrid model would allow usage data to provide feedback to local clinics and for the generated plans to be reviewed periodically by professionals. In communities with sporadic medical visits, monthly access to the kiosk could promote self-management and, in the long term, be reflected in better clinical indicators.

### Limitations

The findings confirm that the kiosk is a viable tool for improving nutrition education. Its low cost and offline capability make it ideal for rural areas; however, it still has some limitations. The AI model was trained with synthetic data, so it requires a second training phase with real data to verify its generalizability.

Furthermore, the high levels of digital literacy among the evaluators could influence the high values of the usability metrics. However, as proposed by Nielsen and Molich [19], the participation of specialists with a technical background is appropriate in a heuristic phase, which should not be considered a bias, but rather a valid methodological criterion for this phase of the research.

Another limitation is that the system focuses on the nutritional component of diabetes treatment, while neglecting other important components such as medication and exercise. These elements will be considered in future versions.

The architecture is adaptable, but the nutritional framework was designed for communities in the northern part of the state of Veracruz, Mexico; therefore, its application to other communities would require adaptations. Despite these limitations, the study represents a promising first step toward creating feasible, culturally appropriate, and sustainable community digital health solutions.

### Recommendations and Future Work

In the short term, a kiosk is expected to be installed in the community of Vega de Otates, Veracruz, Mexico, and a second phase of model training will be carried out using real data from patients with diabetes. This new phase will include incremental and federated learning to improve its performance without compromising user privacy.

The data generated will be used to calibrate the model, so that recommendations can be progressively adapted to demographic, nutritional, and socioeconomic profiles for applicability in other communities. Furthermore, the plan is to integrate continuous glucose monitoring with wearables and AI algorithms and expand the system with personalized physical activity tips and medication reminders. This will allow progress toward a personalized medicine approach, with real-time data collection and dynamic adaptation of nutritional recommendations through automatic pattern recognition.

Finally, a longitudinal field study is planned to evaluate usage indicators, user perceptions, changes in weight, and glycated hemoglobin levels to assess the system’s real impact on health.

### Conclusions

An AI-based interactive kiosk was designed, developed, and technically validated to provide personalized nutritional recommendations. The model achieved satisfactory performance, with an accuracy of 87.3%, precision of 90.5%, sensitivity of 92.1%, and an average *F*_1_-score of 91.3%. The results show a correlation between coverage and precision. In the usability evaluation, the software achieved a mean score of 89.17 (SD 2.89) points on the SUS scale (grade A) and a success rate of 98.3%. Its free, offline, and adaptable nature makes it an innovative tool for promoting nutrition education and reducing health inequalities. The software features an AI recommendation engine, a user-friendly interface, and a local nutritional database with response times of 1.25 to 4 seconds, ensuring its accessibility. The results show that AI technologies can support nutritional self-management. Implementation of the kiosk and its validation in a community pilot study are planned. This project represents a step toward the equitable digitization of nutritional care, establishing and offering the scientific community a replicable, scalable, and sustainable model for public health for communities in Latin America (Multimedia Appendix 1).

## Supplementary material

10.2196/76936 Multimedia Appendix 1 Statistical analyses.

10.2196/76936 Checklist 1 STARE-HI checklist.

## Abbreviations

AI: artificial intelligence
ENSANUT: Encuesta Nacional de Salud y Nutrición Continua
STARE-HI: Statement on Reporting of Evaluation Studies in Health Informatics
SUS: System Usability Scale
UI: user interface
