# Supplementary material for: Smart Kiosk for Nutritional Management of People With Diabetes in Underserved Communities: Development and Technical Evaluation
Source: JMIR Form Res. 2026 Feb 19;10:e76936. doi: 10.2196/76936 (PMC12919750; doi:10.2196/76936)
Supplement: Checklist 1 [file formative-v10-e76936-s002.pdf]

Multimedia Appendix 1 – STARE-HI Checklist

Statement on Reporting of Evaluation Studies in Health Informatics

STARE-HI Item	Description	Location in Manuscript
TITLE & ABSTRACT		
1. Title	Identifies the document as a health informatics evaluation study.	Title page
2. Abstract	Structured summary including background, methods, results, and conclusions.	Abstract
BACKGROUND		
3. Background and Rationale	Describes the public health need, context, and motivation.	Introduction (paragraphs 1–7)
4. Study Objective	States the purpose and aims of the study.	End of Introduction
METHODS – SYSTEM DESCRIPTION		
5. Study Context / Setting	Describes intended environment and population.	Introduction; Methods – Overall Design
6. Description of Informatics System	Architecture, AI model, UI, database, components.	Methods – Phase 1 (Development)
METHODS – EVALUATION DESIGN		
7. Study Design	Identifies evaluation as technical/experimental.	Methods – Overall Research Design
8. Participants or Data Sources	Synthetic data description; no human subjects.	Methods – Phase 2; Ethical Considerations
9. Data Collection Methods	Procedures for obtaining model input and system metrics.	Methods – Phases 2 and 3
10. Data Quality and Validity	Filtering, validation, stratification, consistency checks.	Methods – Phase 2
11. Evaluation Procedures	Description of AI testing, load testing, usability evaluation.	Methods – Phase 3
12. Outcome Measures	AI metrics, SUS score, system performance metrics.	Methods – Evaluation Metrics
13. Statistical Methods	Means, SD, confidence intervals, comparisons.	Methods – Phase 3
ETHICS		

14. Ethical Considerations	No human subjects; synthetic data; privacy.	Ethical Considerations
RESULTS		
15. Results – System Performance	AI model performance and classification accuracy.	Results – Performance of AI Model
16. Results – Technical Efficiency	Response time, resource usage, scalability.	Results – System Performance and Efficiency
17. Results – Usability	SUS results, success rate, heuristic evaluation.	Results – Usability
DISCUSSION		
18. Interpretation / Discussion	Interpretation of findings and implications.	Discussion – Principal Findings
19. Limitations	Constraints of synthetic data, generalizability.	Discussion – Limitations
20. Generalizability / Future Work	Application in real settings, upcoming pilot.	Discussion – Recommendations & Future Work
OTHER SECTIONS		
21. Conclusions	Summary of findings and relevance.	Conclusions
22. Data Availability	Public repository link.	Data Availability section
23. Funding	No external funding.	Funding Statement
24. Conflicts of Interest	None declared.	Conflicts of Interest
25. Generative AI Disclosure	Indicates AI use or non-use during writing.	AI Use Disclosure
